# (4a*S*,10a*S*)-7-Hydr­oxy-8-isopropyl-1,1,4a-trimethyl-1,2,3,4,4a,9,10,10a-octa­hydro­phenanthrene: a new diterpenoid compound

**DOI:** 10.1107/S1600536808004546

**Published:** 2008-02-20

**Authors:** Abdellah Zeroual, Noureddine Mazoir, Jean-Claude Daran, Mohamed Akssira, Ahmed Benharref

**Affiliations:** a Laboratoire de Chimie Biomoléculaire, Substances Naturelles et Réactivité, Faculté des Sciences Semlalia, Université Cadi Ayyad, BP 2390 Marrakech, Morocco; b Laboratoire de Chimie de Coordination, 205 route de Narbonne, 31077 Toulouse Cedex 04, France; c Laboratoire de Chimie Bioorganique et Analytique, Faculté des Sciences et Techniques, Université Hassan II-Mohammedia, BP 146-20800 Mohammedia, Morocco

## Abstract

The new title diterpenoid compound, C_20_H_30_O, is a natural product isolated from *Tetra­clinis articulata* wood *via* chloro­form extraction. The asymmetric unit contains four mol­ecules with the same *S*,*S* configuration, deduced from the chemical synthesis. Indeed, an overlay analysis, calculated using structure-matching software, shows that the four mol­ecules can be superimposed. The central ring has a half-chair conformation, whereas the saturated ring displays a chair conformation.

## Related literature

For related literature, see: Barrero *et al.* (2003 ▶); Collins *et al.* (2006 ▶); Cremer & Pople (1975 ▶); Duan *et al.* (2001 ▶); Hedden & Philips (2000 ▶); Rundle *et al.* (2001 ▶); Betteridge *et al.* (2003 ▶); Yang *et al.* (2002 ▶); Zeroual, Mazoir, Berraho *et al.* (2007 ▶); Zeroual, Mazoir, Maya *et al.* (2007 ▶).
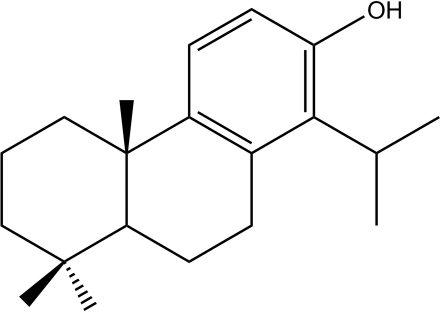


## Experimental

### 

#### Crystal data

C_20_H_30_O
*M* *_r_* = 286.44
Triclinic, 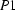

*a* = 10.5422 (5) Å
*b* = 12.1343 (5) Å
*c* = 14.8245 (6) Å
α = 70.578 (4)°
β = 70.096 (4)°
γ = 89.968 (3)°
*V* = 1668.01 (14) Å^3^
*Z* = 4
Mo *K*α radiation
μ = 0.07 mm^−1^
*T* = 180 (2) K
0.48 × 0.40 × 0.22 mm

#### Data collection

Oxford Diffraction Xcalibur diffractometer
Absorption correction: none
13146 measured reflections
6812 independent reflections
4589 reflections with *I* > 2σ(*I*)
*R*_int_ = 0.028

#### Refinement

*R*[*F* ^2^ > 2σ(*F* ^2^)] = 0.040
*wR*(*F* ^2^) = 0.109
*S* = 0.98
6812 reflections
781 parameters
3 restraints
H-atom parameters constrained
Δρ_max_ = 0.16 e Å^−3^
Δρ_min_ = −0.21 e Å^−3^

### 

Data collection: *CrysAlis CCD* (Oxford Diffraction, 2006 ▶); cell refinement: *CrysAlis RED* (Oxford Diffraction, 2006 ▶); data reduction: *CrysAlis RED*; program(s) used to solve structure: *SIR97* (Altomare *et al.*, 1999 ▶); program(s) used to refine structure: *SHELXL97* (Sheldrick, 2008 ▶); molecular graphics: *ORTEPIII* (Burnett & Johnson, 1996 ▶) and *ORTEP-3 for Windows* (Farrugia, 1997 ▶); software used to prepare material for publication: *WinGX* (Farrugia, 1999 ▶).

## Supplementary Material

Crystal structure: contains datablocks I, global. DOI: 10.1107/S1600536808004546/hg2379sup1.cif

Structure factors: contains datablocks I. DOI: 10.1107/S1600536808004546/hg2379Isup2.hkl

Additional supplementary materials:  crystallographic information; 3D view; checkCIF report

## Figures and Tables

**Table 1:** Puckering amplitudes (Cremer & Pople, 1975 ▶) for the non-benzenoid rings in the four independent molecules

	C1/C2/C3/C4/C4a/C10a			C4a/C4b/C8a/C9/C10/C10a		
Molecule	*Q* (Å)	θ (°)	ϕ (°)	*Q* (Å)	θ (°)	ϕ (°)
1	0.553 (3)	4.2 (3)	140 (5)	0.553 (3)	52.3 (3)	284.7 (4)
2	0.550 (3)	6.8 (3)	141 (3)	0.555 (3)	51.9 (3)	289.8 (4)
3	0.548 (4)	7.9 (4)	142 (3)	0.543 (3)	51.0 (3)	296.2 (4)
4	0.547 (4)	6.6 (4)	139 (3)	0.543 (3)	51.0 (3)	289.2 (4)

**Table 2:** Structure matching (Å, °) between the four independent molecules *A* is the structure match between molecules 1 and 2, *B* between molecules 1 and 3, *C* between molecules 1 and 4, *D* between molecules 2 and 3, *E* between molecules 2 and 4, and *F* between molecules 3 and 4.

Overlay	r.m.s. bond length	r.m.s. torsion angle	r.m.s. torsion
*A*	0.0707	0.0074	2.1002
*B*	0.1754	0.0074	5.0807
*C*	0.0917	0.0073	2.7283
*D*	0.1174	0.0063	3.479
*E*	0.0505	0.0093	1.5649
*F*	0.0896	0.0091	2.6273

**Fig. 1.**
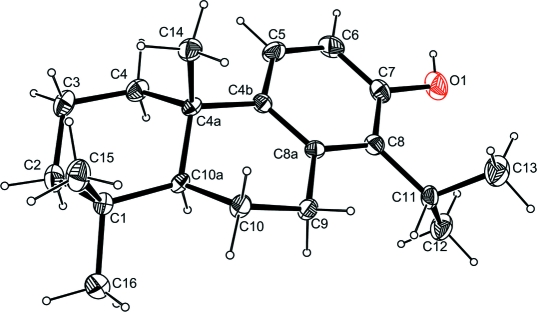
Molecular view of one of the four molecules of the title compound with the atom-labelling scheme. Ellipsoids are drawn at the 30% probability level. H atoms are represented as small spheres of arbitrary radii.

## supplementary crystallographic information

### Comment

Among tricyclic diterpenoids, the class based on the phenanthrene skeleton is of great interest in alimentary, agricultural and cosmetic industries. Many of these substances exhibit biological activities that have been recently reported (Hedden & Philips, 2000; Duan *et al.*, 2001; Rundle *et al.*, 2001; Yang *et al.*, 2002).

Within the context of our research for the chemical constituents of *Tetraclinis articulata *(Barrero *et al.*, 2003), we have isolated two components structurally related to the diterpenoid skeletons (Zeroual, Mazoir, Berraho *et al.* (2007); Zeroual, Mazoir, Maya *et al.* (2007). The title compound (I), was isolated from *Tetraclinis articulata* wood using extraction with chloroform in a sohxlet apparatus.

The structure of (I) was established by ^1^H and ^13^C NMR and confirmed by its single-Crystal X-ray structure. The unit cell in space group P1 contains four identical molecules having the same configuration (*S,S*). Indeed an overlay analyses calculated using the structure matching software (Watkin *et al.*, 2003; Collins *et al.*, 2006) shows that the four molecules could be superimposed (Table 1).

Each of these molecules is built up from three six-membered fused rings, a saturated one and two unsaturated (Fig. 1). The central rings display a half-chair conformation whereas the other unsaturated six-membered ring has a chair conformation (Cremer & Pople, 1975; Table 2).

### Experimental

50 g of *Tetraclinis articulata* wood was extracted with chloroform (300 ml) in a Sohxlet apparatus during 24 h. The CHCl_3_ solution was cooled to yield, after solvent removal, one fraction (3.2 g) which was then subjected to silica gel column chromatography using hexane as an eluent afforded compound (I) in 64% yield. Suitable crystals of (I) were obtained by evaporation of a hexane solution at 277 K. m.p. = 373–374 K (hexane); Spectroscopic analysis: ^1^H NMR (300 MHz, CDCl_3_, δ, p.p.m.): 1.51 (2H2, m), 1.58 (2H3, m), 1.60 (2H4, m), 7.02 (1H5, d, J = 8.7 Hz), 6.60 (1H6, d, J = 8.7 Hz), 4.50 (OH, s), 2.84 (2H9, m), 1.58 (2H10, m), 1.76 (1H10*a*, dd, J_1_ = 10.6 Hz, J_2_ = 2.0 Hz), 3.11 (1H11, m), 1.30 (3H12, d, J = 10 Hz), 1.31 (3H13, d, J = 10 Hz), 0.97 (3H14, s), 0.98 (3H15, s), 1.11 (3H16, s); ^13^C NMR (75 MHz, CDCl_3_, d, p.p.m.): 37.8 (C1), 41.6 (C2), 19.6 (C3), 39.6 (C4),37.5 (C4a), 131.1 (C4b), 123.0 (C5), 114.4 (C6), 152.1 (C7), 143.3 (C8), 134.1(C8a), 28.8 (C9), 19.5 (C10), 49.6 (C10*a*), 33.3 (C11), 20.1 (C12), 20.2 (C13), 22.4 (C14), 25.5 (C15), 24.6 (C16).

### Refinement

All H atoms attached to C and O atoms were fixed geometrically and treated as riding with C—H = 0.99 Å (methyl), 0.98 Å (methylene), 1.0 Å(methine) or 0.95 Å (aromatic) and O—H = 0.84 Å with *U*_iso_(H) = 1.2*U*_eq_(C or O) or *U*_iso_(H) = 1.5*U*_eq_(methyl).

In the absence of significant anomalous scattering, the absolute configuration could not determined by X-ray analyses and then the Friedel pairs were merged and any references to the Flack parameter were removed. The absolute configuration was deduced from the chemical syntheses.

### Crystal data

**Table:**

C_20_H_30_O_1_	*Z* = 4
*M**_r_* = 286.44	*F*_000_ = 632
Triclinic, *P*1	*D*_x_ = 1.141 Mg m^−^^3^
Hall symbol: P 1	Mo *K*α radiation λ = 0.71073 Å
*a* = 10.5422 (5) Å	Cell parameters from 6230 reflections
*b* = 12.1343 (5) Å	θ = 2.8–32.1º
*c* = 14.8245 (6) Å	µ = 0.07 mm^−^^1^
α = 70.578 (4)º	*T* = 180 (2) K
β = 70.096 (4)º	Block, colourless
γ = 89.968 (3)º	0.48 × 0.40 × 0.22 mm
*V* = 1668.01 (14) Å^3^	

### Data collection

**Table:**

Oxford Diffraction Xcalibur diffractometer	6812 independent reflections
Radiation source: fine-focus sealed tube	4589 reflections with *I* > 2σ(*I*)
Monochromator: graphite	*R*_int_ = 0.028
Detector resolution: 8.2632 pixels mm^-1^	θ_max_ = 26.4º
*T* = 180(2) K	θ_min_ = 2.8º
ω and φ scans	*h* = −13→13
Absorption correction: none	*k* = −14→15
13146 measured reflections	*l* = −16→18

### Refinement

**Table:**

Refinement on *F*^2^	Secondary atom site location: difference Fourier map
Least-squares matrix: full	Hydrogen site location: inferred from neighbouring sites
*R*[*F*^2^ > 2σ(*F*^2^)] = 0.040	H-atom parameters constrained
*wR*(*F*^2^) = 0.109	*w* = 1/[σ^2^(*F*_o_^2^) + (0.0625*P*)^2^] where *P* = (*F*_o_^2^ + 2*F*_c_^2^)/3
*S* = 0.98	(Δ/σ)_max_ = 0.001
6812 reflections	Δρ_max_ = 0.16 e Å^−^^3^
781 parameters	Δρ_min_ = −0.21 e Å^−^^3^
3 restraints	Extinction correction: none
Primary atom site location: structure-invariant direct methods	

### Special details

**Table:**

Geometry. All e.s.d.'s (except the e.s.d. in the dihedral angle between two l.s. planes) are estimated using the full covariance matrix. The cell e.s.d.'s are taken into account individually in the estimation of e.s.d.'s in distances, angles and torsion angles; correlations between e.s.d.'s in cell parameters are only used when they are defined by crystal symmetry. An approximate (isotropic) treatment of cell e.s.d.'s is used for estimating e.s.d.'s involving l.s. planes.
Refinement. Refinement of *F*^2^ against ALL reflections. The weighted *R*-factor *wR* and goodness of fit *S* are based on *F*^2^, conventional *R*-factors *R* are based on *F*, with *F* set to zero for negative *F*^2^. The threshold expression of *F*^2^ > σ(*F*^2^) is used only for calculating *R*-factors(gt) *etc*. and is not relevant to the choice of reflections for refinement. *R*-factors based on *F*^2^ are statistically about twice as large as those based on *F*, and *R*- factors based on ALL data will be even larger.

### Fractional atomic coordinates and isotropic or equivalent isotropic displacement parameters (Å^2^)

**Table:**

	*x*	*y*	*z*	*U*_iso_*/*U*_eq_	
O1	0.1167 (3)	0.09842 (18)	0.96490 (17)	0.0523 (7)	
H1	0.0802	0.0758	1.0291	0.078*	
C1	0.3301 (3)	0.7807 (2)	0.6414 (2)	0.0315 (7)	
C2	0.3671 (3)	0.8211 (3)	0.7172 (2)	0.0386 (8)	
H2A	0.4513	0.7893	0.7237	0.046*	
H2B	0.3862	0.9082	0.6896	0.046*	
C3	0.2567 (3)	0.7828 (3)	0.8224 (2)	0.0404 (8)	
H3A	0.1746	0.8200	0.8174	0.048*	
H3B	0.2880	0.8097	0.8684	0.048*	
C4	0.2208 (3)	0.6491 (3)	0.8680 (2)	0.0349 (7)	
H4A	0.1477	0.6272	0.9364	0.042*	
H4B	0.3014	0.6125	0.8775	0.042*	
C4A	0.1733 (3)	0.5998 (2)	0.80008 (19)	0.0231 (6)	
C4B	0.1609 (3)	0.4646 (2)	0.8406 (2)	0.0243 (6)	
C5	0.1037 (3)	0.4046 (3)	0.9454 (2)	0.0365 (8)	
H5	0.0740	0.4483	0.9898	0.044*	
C6	0.0887 (3)	0.2847 (3)	0.9868 (2)	0.0425 (8)	
H6	0.0486	0.2460	1.0590	0.051*	
C7	0.1316 (3)	0.2202 (3)	0.9243 (2)	0.0350 (7)	
C8	0.1919 (3)	0.2750 (2)	0.8182 (2)	0.0280 (7)	
C8A	0.2012 (3)	0.3984 (2)	0.7770 (2)	0.0239 (6)	
C9	0.2569 (3)	0.4581 (2)	0.6617 (2)	0.0285 (7)	
H9A	0.2076	0.4191	0.6321	0.034*	
H9B	0.3538	0.4467	0.6354	0.034*	
C10	0.2455 (3)	0.5897 (2)	0.6247 (2)	0.0298 (7)	
H10B	0.3070	0.6274	0.5524	0.036*	
H10C	0.1512	0.6019	0.6286	0.036*	
C10A	0.2836 (3)	0.6453 (2)	0.69120 (19)	0.0232 (6)	
H10A	0.3662	0.6098	0.7000	0.028*	
C11	0.2451 (3)	0.2019 (2)	0.7505 (2)	0.0317 (7)	
H11	0.2900	0.2588	0.6788	0.038*	
C12	0.3523 (3)	0.1277 (3)	0.7767 (3)	0.0465 (9)	
H12A	0.3116	0.0685	0.8457	0.070*	
H12B	0.4266	0.1785	0.7742	0.070*	
H12C	0.3880	0.0882	0.7270	0.070*	
C13	0.1324 (4)	0.1245 (3)	0.7495 (3)	0.0537 (10)	
H13A	0.0648	0.1731	0.7306	0.081*	
H13B	0.0887	0.0645	0.8179	0.081*	
H13C	0.1711	0.0859	0.6995	0.081*	
C14	0.0312 (3)	0.6331 (3)	0.8057 (2)	0.0340 (7)	
H14A	−0.0341	0.5931	0.8754	0.051*	
H14B	0.0043	0.6089	0.7573	0.051*	
H14C	0.0327	0.7186	0.7879	0.051*	
C15	0.2264 (4)	0.8541 (3)	0.6058 (3)	0.0441 (8)	
H15A	0.1909	0.8177	0.5678	0.066*	
H15B	0.2706	0.9341	0.5612	0.066*	
H15C	0.1513	0.8574	0.6657	0.066*	
C16	0.4607 (4)	0.8047 (3)	0.5459 (3)	0.0500 (9)	
H16A	0.4998	0.8872	0.5211	0.075*	
H16B	0.4384	0.7889	0.4920	0.075*	
H16C	0.5266	0.7532	0.5641	0.075*	
O2	0.8528 (3)	0.97439 (18)	0.60117 (17)	0.0518 (6)	
H2	0.8989	0.9980	0.5381	0.078*	
C21	0.6785 (3)	0.2891 (2)	0.9123 (2)	0.0320 (7)	
C22	0.7924 (3)	0.2434 (3)	0.8428 (2)	0.0413 (8)	
H22A	0.8803	0.2676	0.8456	0.050*	
H22B	0.7771	0.1562	0.8695	0.050*	
C23	0.8020 (4)	0.2876 (3)	0.7326 (2)	0.0422 (8)	
H23A	0.7167	0.2592	0.7286	0.051*	
H23B	0.8777	0.2555	0.6919	0.051*	
C24	0.8258 (3)	0.4220 (3)	0.6870 (2)	0.0347 (7)	
H24A	0.8327	0.4481	0.6145	0.042*	
H24B	0.9131	0.4501	0.6883	0.042*	
C24A	0.7099 (3)	0.4774 (2)	0.7467 (2)	0.0248 (6)	
C24B	0.7475 (3)	0.6111 (2)	0.7106 (2)	0.0257 (6)	
C25	0.8194 (3)	0.6739 (3)	0.6071 (2)	0.0364 (7)	
H25	0.8447	0.6328	0.5604	0.044*	
C26	0.8542 (3)	0.7929 (3)	0.5712 (2)	0.0431 (9)	
H26	0.9029	0.8339	0.5002	0.052*	
C27	0.8190 (3)	0.8534 (3)	0.6376 (2)	0.0348 (7)	
C28	0.7493 (3)	0.7974 (2)	0.7412 (2)	0.0258 (6)	
C28A	0.7106 (3)	0.6744 (2)	0.7776 (2)	0.0238 (6)	
C29	0.6309 (3)	0.6123 (2)	0.8901 (2)	0.0299 (7)	
H29A	0.5509	0.6536	0.9105	0.036*	
H29B	0.6885	0.6193	0.9291	0.036*	
C30	0.5816 (3)	0.4823 (2)	0.9215 (2)	0.0308 (7)	
H30B	0.4977	0.4742	0.9071	0.037*	
H30C	0.5603	0.4423	0.9960	0.037*	
C30A	0.6923 (3)	0.4254 (2)	0.8615 (2)	0.0249 (6)	
H30A	0.7785	0.4553	0.8646	0.030*	
C211	0.7177 (3)	0.8651 (2)	0.8143 (2)	0.0299 (7)	
H211	0.6740	0.8059	0.8851	0.036*	
C212	0.8445 (3)	0.9267 (3)	0.8120 (3)	0.0511 (9)	
H12D	0.8850	0.9919	0.7464	0.077*	
H12E	0.9102	0.8705	0.8201	0.077*	
H12F	0.8203	0.9575	0.8682	0.077*	
C213	0.6172 (3)	0.9520 (3)	0.7982 (3)	0.0457 (8)	
H13D	0.5317	0.9099	0.8076	0.068*	
H13E	0.6548	1.0104	0.7286	0.068*	
H13F	0.6000	0.9919	0.8481	0.068*	
C214	0.5822 (3)	0.4567 (3)	0.7231 (2)	0.0373 (7)	
H14D	0.5993	0.5000	0.6505	0.056*	
H14E	0.5054	0.4847	0.7654	0.056*	
H14F	0.5608	0.3724	0.7385	0.056*	
C215	0.5373 (3)	0.2246 (3)	0.9368 (3)	0.0485 (9)	
H15D	0.5341	0.1402	0.9733	0.073*	
H15E	0.5224	0.2360	0.8727	0.073*	
H15F	0.4663	0.2567	0.9799	0.073*	
C216	0.7010 (4)	0.2589 (3)	1.0143 (2)	0.0470 (9)	
H16D	0.7864	0.3028	1.0029	0.070*	
H16E	0.7056	0.1742	1.0421	0.070*	
H16F	0.6253	0.2803	1.0632	0.070*	
O3	0.8840 (3)	0.9285 (2)	0.07938 (18)	0.0581 (7)	
H3	0.9311	0.9464	0.0169	0.087*	
C31	0.6454 (3)	0.2602 (2)	0.4319 (2)	0.0359 (7)	
C32	0.7527 (3)	0.2014 (3)	0.3711 (2)	0.0432 (8)	
H32A	0.8417	0.2220	0.3739	0.052*	
H32B	0.7290	0.1149	0.4042	0.052*	
C33	0.7665 (3)	0.2360 (3)	0.2608 (2)	0.0411 (8)	
H33A	0.6796	0.2112	0.2568	0.049*	
H33B	0.8379	0.1953	0.2260	0.049*	
C34	0.8037 (3)	0.3691 (3)	0.2065 (2)	0.0352 (7)	
H34A	0.8147	0.3897	0.1336	0.042*	
H34B	0.8919	0.3929	0.2090	0.042*	
C34A	0.6952 (3)	0.4374 (2)	0.2557 (2)	0.0261 (6)	
C34B	0.7445 (3)	0.5702 (2)	0.2100 (2)	0.0264 (7)	
C35	0.8292 (3)	0.6205 (3)	0.1079 (2)	0.0401 (8)	
H35	0.8560	0.5723	0.0674	0.048*	
C36	0.8749 (4)	0.7386 (3)	0.0648 (2)	0.0481 (9)	
H36	0.9332	0.7712	−0.0051	0.058*	
C37	0.8372 (3)	0.8099 (3)	0.1215 (2)	0.0379 (8)	
C38	0.7512 (3)	0.7654 (2)	0.2229 (2)	0.0290 (7)	
C38A	0.7050 (3)	0.6438 (2)	0.2673 (2)	0.0244 (6)	
C39	0.6157 (3)	0.5934 (2)	0.3800 (2)	0.0333 (7)	
H39A	0.5361	0.6374	0.3911	0.040*	
H39B	0.6676	0.6073	0.4204	0.040*	
C40A	0.6711 (3)	0.3950 (2)	0.3713 (2)	0.0264 (6)	
H40A	0.7576	0.4230	0.3753	0.032*	
C40	0.5640 (3)	0.4620 (2)	0.4216 (2)	0.0327 (7)	
H40B	0.5414	0.4303	0.4968	0.039*	
H40C	0.4801	0.4508	0.4081	0.039*	
C311	0.7120 (3)	0.8454 (3)	0.2858 (3)	0.0394 (8)	
H311	0.6449	0.7958	0.3544	0.047*	
C312	0.8296 (4)	0.8890 (4)	0.3053 (3)	0.0646 (12)	
H12G	0.8986	0.9383	0.2400	0.097*	
H12H	0.8692	0.8217	0.3389	0.097*	
H12I	0.7979	0.9355	0.3497	0.097*	
C313	0.6415 (4)	0.9483 (3)	0.2425 (4)	0.0693 (13)	
H13G	0.5665	0.9192	0.2281	0.104*	
H13H	0.7068	1.0046	0.1789	0.104*	
H13I	0.6057	0.9874	0.2925	0.104*	
C314	0.5678 (3)	0.4218 (3)	0.2302 (2)	0.0392 (8)	
H14G	0.5363	0.3377	0.2533	0.059*	
H14H	0.5903	0.4572	0.1559	0.059*	
H14I	0.4958	0.4606	0.2651	0.059*	
C315	0.5018 (3)	0.2012 (3)	0.4569 (3)	0.0465 (9)	
H15G	0.4901	0.1190	0.5024	0.070*	
H15H	0.4907	0.2037	0.3933	0.070*	
H15I	0.4333	0.2434	0.4910	0.070*	
C316	0.6655 (4)	0.2384 (3)	0.5336 (2)	0.0543 (10)	
H16G	0.6617	0.1536	0.5686	0.081*	
H16H	0.5935	0.2694	0.5771	0.081*	
H16I	0.7543	0.2783	0.5204	0.081*	
O4	0.1205 (2)	0.05884 (18)	0.44649 (17)	0.0487 (6)	
H4	0.1159	0.0322	0.5077	0.073*	
C41	0.3018 (3)	0.7513 (2)	0.1661 (2)	0.0337 (7)	
C42	0.3250 (3)	0.7861 (3)	0.2501 (3)	0.0434 (8)	
H42A	0.4099	0.7576	0.2583	0.052*	
H42B	0.3378	0.8731	0.2278	0.052*	
C43	0.2094 (4)	0.7374 (3)	0.3533 (3)	0.0451 (9)	
H43A	0.1255	0.7702	0.3471	0.054*	
H43B	0.2319	0.7620	0.4040	0.054*	
C44	0.1849 (3)	0.6035 (3)	0.3906 (2)	0.0367 (7)	
H44A	0.1092	0.5741	0.4579	0.044*	
H44B	0.2674	0.5710	0.4003	0.044*	
C44A	0.1501 (3)	0.5599 (2)	0.3150 (2)	0.0252 (6)	
C44B	0.1458 (3)	0.4254 (2)	0.3473 (2)	0.0254 (6)	
C45	0.0939 (3)	0.3564 (3)	0.4506 (2)	0.0406 (8)	
H45	0.0636	0.3937	0.5000	0.049*	
C46	0.0850 (3)	0.2350 (3)	0.4839 (2)	0.0435 (9)	
H46	0.0479	0.1895	0.5551	0.052*	
C47	0.1301 (3)	0.1814 (3)	0.4134 (2)	0.0345 (7)	
C48	0.1831 (3)	0.2444 (2)	0.3093 (2)	0.0285 (7)	
C48A	0.1886 (3)	0.3683 (2)	0.2761 (2)	0.0248 (6)	
C49	0.2438 (3)	0.4377 (2)	0.1625 (2)	0.0300 (7)	
H49A	0.1953	0.4039	0.1291	0.036*	
H49B	0.3411	0.4281	0.1345	0.036*	
C50	0.2309 (3)	0.5683 (2)	0.1341 (2)	0.0312 (7)	
H50B	0.2948	0.6113	0.0634	0.037*	
H50C	0.1374	0.5813	0.1361	0.037*	
C50A	0.2621 (3)	0.6154 (2)	0.2079 (2)	0.0270 (6)	
H50A	0.3460	0.5814	0.2154	0.032*	
C411	0.2381 (3)	0.1805 (2)	0.2333 (2)	0.0344 (7)	
H411	0.2705	0.2420	0.1630	0.041*	
C412	0.3594 (3)	0.1187 (3)	0.2459 (3)	0.0468 (9)	
H12J	0.3956	0.0851	0.1920	0.070*	
H12K	0.3310	0.0557	0.3132	0.070*	
H12L	0.4300	0.1756	0.2406	0.070*	
C413	0.1285 (4)	0.0942 (3)	0.2377 (3)	0.0526 (10)	
H13J	0.1045	0.0258	0.3014	0.079*	
H13K	0.1631	0.0682	0.1788	0.079*	
H13L	0.0476	0.1333	0.2358	0.079*	
C414	0.0053 (3)	0.5861 (3)	0.3207 (2)	0.0358 (7)	
H14J	−0.0590	0.5453	0.3907	0.054*	
H14K	−0.0192	0.5585	0.2730	0.054*	
H14L	0.0018	0.6711	0.3021	0.054*	
C415	0.1991 (4)	0.8224 (3)	0.1279 (2)	0.0451 (9)	
H15J	0.1796	0.7938	0.0789	0.068*	
H15K	0.2368	0.9058	0.0942	0.068*	
H15L	0.1149	0.8133	0.1861	0.068*	
C416	0.4386 (4)	0.7830 (3)	0.0761 (3)	0.0577 (11)	
H16J	0.4717	0.8663	0.0558	0.087*	
H16K	0.4266	0.7689	0.0180	0.087*	
H16L	0.5048	0.7341	0.0971	0.087*	

### Atomic displacement parameters (Å^2^)

**Table:**

	*U*^11^	*U*^22^	*U*^33^	*U*^12^	*U*^13^	*U*^23^
O1	0.0690 (18)	0.0265 (12)	0.0444 (14)	−0.0080 (11)	−0.0136 (13)	0.0012 (11)
C1	0.0334 (18)	0.0237 (16)	0.0343 (17)	0.0028 (13)	−0.0072 (14)	−0.0116 (13)
C2	0.0403 (19)	0.0297 (17)	0.053 (2)	0.0025 (14)	−0.0212 (16)	−0.0187 (15)
C3	0.050 (2)	0.0388 (19)	0.046 (2)	0.0084 (15)	−0.0215 (17)	−0.0271 (16)
C4	0.0388 (18)	0.0408 (18)	0.0320 (17)	0.0077 (14)	−0.0142 (14)	−0.0202 (14)
C4A	0.0229 (15)	0.0256 (15)	0.0246 (15)	0.0092 (12)	−0.0100 (12)	−0.0120 (12)
C4B	0.0227 (15)	0.0256 (15)	0.0249 (16)	0.0017 (12)	−0.0095 (12)	−0.0083 (13)
C5	0.0428 (19)	0.0385 (19)	0.0252 (17)	0.0061 (15)	−0.0056 (14)	−0.0143 (14)
C6	0.046 (2)	0.042 (2)	0.0223 (17)	−0.0045 (15)	−0.0025 (15)	−0.0008 (15)
C7	0.0370 (18)	0.0240 (17)	0.0361 (19)	−0.0018 (14)	−0.0120 (15)	−0.0019 (14)
C8	0.0267 (16)	0.0258 (16)	0.0307 (17)	0.0037 (12)	−0.0106 (13)	−0.0086 (13)
C8A	0.0212 (15)	0.0265 (16)	0.0238 (15)	0.0040 (12)	−0.0080 (12)	−0.0089 (12)
C9	0.0370 (17)	0.0240 (15)	0.0244 (15)	0.0050 (12)	−0.0092 (13)	−0.0103 (12)
C10	0.0418 (18)	0.0241 (15)	0.0232 (15)	0.0060 (13)	−0.0118 (13)	−0.0078 (12)
C10A	0.0272 (16)	0.0228 (14)	0.0242 (15)	0.0101 (12)	−0.0111 (13)	−0.0119 (12)
C11	0.0353 (18)	0.0188 (15)	0.0413 (18)	0.0027 (12)	−0.0130 (14)	−0.0118 (13)
C12	0.041 (2)	0.044 (2)	0.069 (2)	0.0158 (16)	−0.0246 (18)	−0.0334 (18)
C13	0.046 (2)	0.060 (2)	0.076 (3)	0.0112 (18)	−0.033 (2)	−0.039 (2)
C14	0.0277 (16)	0.0359 (17)	0.0420 (18)	0.0090 (13)	−0.0134 (14)	−0.0175 (14)
C15	0.060 (2)	0.0293 (17)	0.046 (2)	0.0110 (16)	−0.0255 (17)	−0.0111 (15)
C16	0.047 (2)	0.0361 (19)	0.051 (2)	−0.0003 (15)	−0.0007 (17)	−0.0143 (16)
O2	0.0609 (16)	0.0286 (13)	0.0434 (14)	−0.0068 (11)	−0.0008 (12)	−0.0033 (11)
C21	0.0470 (19)	0.0214 (15)	0.0352 (17)	0.0072 (13)	−0.0218 (15)	−0.0122 (13)
C22	0.057 (2)	0.0292 (17)	0.048 (2)	0.0200 (15)	−0.0255 (17)	−0.0186 (15)
C23	0.055 (2)	0.0405 (19)	0.043 (2)	0.0218 (16)	−0.0192 (16)	−0.0271 (16)
C24	0.0419 (19)	0.0368 (18)	0.0296 (17)	0.0160 (14)	−0.0118 (14)	−0.0182 (14)
C24A	0.0285 (16)	0.0235 (15)	0.0255 (15)	0.0094 (12)	−0.0107 (13)	−0.0114 (12)
C24B	0.0273 (16)	0.0247 (15)	0.0257 (16)	0.0054 (12)	−0.0113 (13)	−0.0076 (13)
C25	0.047 (2)	0.0361 (18)	0.0234 (16)	0.0052 (15)	−0.0076 (14)	−0.0123 (14)
C26	0.049 (2)	0.041 (2)	0.0228 (17)	0.0005 (16)	−0.0025 (15)	−0.0022 (15)
C27	0.0365 (18)	0.0248 (16)	0.0328 (18)	−0.0020 (13)	−0.0065 (14)	−0.0036 (14)
C28	0.0243 (15)	0.0213 (15)	0.0312 (17)	0.0019 (12)	−0.0107 (13)	−0.0077 (13)
C28A	0.0227 (15)	0.0226 (15)	0.0265 (15)	0.0036 (12)	−0.0102 (12)	−0.0079 (12)
C29	0.0349 (17)	0.0243 (15)	0.0276 (16)	0.0058 (13)	−0.0048 (13)	−0.0123 (13)
C30	0.0360 (17)	0.0215 (15)	0.0282 (16)	0.0017 (13)	−0.0037 (13)	−0.0086 (13)
C30A	0.0311 (16)	0.0201 (14)	0.0240 (15)	0.0022 (12)	−0.0095 (13)	−0.0089 (12)
C211	0.0338 (17)	0.0215 (15)	0.0372 (17)	0.0045 (12)	−0.0149 (14)	−0.0118 (13)
C212	0.044 (2)	0.054 (2)	0.078 (3)	0.0183 (17)	−0.0318 (19)	−0.041 (2)
C213	0.0367 (19)	0.045 (2)	0.065 (2)	0.0143 (15)	−0.0224 (17)	−0.0269 (18)
C214	0.0428 (19)	0.0368 (18)	0.0455 (19)	0.0095 (14)	−0.0291 (15)	−0.0173 (15)
C215	0.059 (2)	0.0337 (19)	0.050 (2)	−0.0116 (17)	−0.0182 (18)	−0.0118 (16)
C216	0.071 (2)	0.0310 (18)	0.040 (2)	0.0073 (16)	−0.0253 (18)	−0.0076 (15)
O3	0.0732 (19)	0.0369 (13)	0.0404 (14)	−0.0227 (12)	−0.0078 (13)	0.0025 (11)
C31	0.049 (2)	0.0244 (16)	0.0346 (18)	0.0052 (14)	−0.0157 (15)	−0.0101 (14)
C32	0.050 (2)	0.0289 (17)	0.056 (2)	0.0148 (15)	−0.0251 (17)	−0.0153 (16)
C33	0.0430 (19)	0.0364 (19)	0.049 (2)	0.0137 (15)	−0.0133 (16)	−0.0242 (16)
C34	0.0371 (18)	0.0391 (19)	0.0335 (17)	0.0076 (14)	−0.0095 (14)	−0.0213 (15)
C34A	0.0297 (16)	0.0264 (15)	0.0277 (16)	0.0052 (12)	−0.0146 (13)	−0.0123 (13)
C34B	0.0263 (16)	0.0287 (16)	0.0240 (16)	0.0016 (12)	−0.0109 (13)	−0.0073 (13)
C35	0.051 (2)	0.043 (2)	0.0251 (17)	−0.0014 (16)	−0.0099 (15)	−0.0149 (15)
C36	0.056 (2)	0.048 (2)	0.0235 (18)	−0.0130 (17)	−0.0019 (16)	−0.0049 (16)
C37	0.044 (2)	0.0277 (17)	0.0319 (19)	−0.0099 (15)	−0.0132 (15)	0.0012 (15)
C38	0.0258 (16)	0.0267 (16)	0.0353 (18)	0.0041 (12)	−0.0134 (14)	−0.0094 (14)
C38A	0.0178 (14)	0.0281 (16)	0.0259 (15)	0.0047 (12)	−0.0083 (12)	−0.0073 (13)
C39	0.0329 (17)	0.0253 (16)	0.0330 (17)	0.0047 (13)	0.0000 (14)	−0.0117 (13)
C40A	0.0274 (16)	0.0249 (15)	0.0272 (16)	0.0059 (12)	−0.0087 (13)	−0.0109 (13)
C40	0.0318 (17)	0.0270 (16)	0.0302 (16)	0.0010 (13)	−0.0010 (13)	−0.0094 (13)
C311	0.039 (2)	0.0181 (16)	0.047 (2)	0.0015 (13)	−0.0045 (16)	−0.0055 (14)
C312	0.077 (3)	0.075 (3)	0.087 (3)	0.045 (2)	−0.056 (2)	−0.058 (2)
C313	0.055 (2)	0.037 (2)	0.142 (4)	0.0200 (18)	−0.061 (3)	−0.039 (2)
C314	0.0421 (19)	0.0372 (18)	0.049 (2)	0.0082 (14)	−0.0263 (16)	−0.0179 (16)
C315	0.049 (2)	0.0277 (18)	0.049 (2)	−0.0077 (15)	−0.0050 (17)	−0.0097 (15)
C316	0.082 (3)	0.036 (2)	0.041 (2)	0.0088 (18)	−0.0267 (19)	−0.0034 (16)
O4	0.0583 (15)	0.0266 (12)	0.0436 (14)	0.0055 (11)	−0.0105 (13)	0.0013 (10)
C41	0.0371 (18)	0.0245 (16)	0.0375 (18)	0.0056 (13)	−0.0079 (14)	−0.0141 (14)
C42	0.049 (2)	0.0264 (17)	0.065 (2)	0.0059 (15)	−0.0290 (18)	−0.0201 (16)
C43	0.070 (2)	0.0374 (19)	0.046 (2)	0.0100 (17)	−0.0294 (18)	−0.0284 (16)
C44	0.0460 (19)	0.0383 (18)	0.0364 (18)	0.0137 (15)	−0.0195 (15)	−0.0216 (15)
C44A	0.0276 (16)	0.0233 (15)	0.0272 (16)	0.0067 (12)	−0.0106 (13)	−0.0113 (13)
C44B	0.0260 (16)	0.0280 (16)	0.0239 (15)	0.0051 (12)	−0.0106 (13)	−0.0095 (13)
C45	0.049 (2)	0.041 (2)	0.0278 (18)	0.0073 (16)	−0.0065 (15)	−0.0156 (16)
C46	0.054 (2)	0.0368 (19)	0.0243 (17)	0.0050 (16)	−0.0043 (16)	−0.0014 (15)
C47	0.0353 (18)	0.0238 (16)	0.0343 (18)	0.0059 (13)	−0.0098 (15)	−0.0005 (14)
C48	0.0247 (15)	0.0278 (17)	0.0316 (17)	0.0053 (12)	−0.0075 (13)	−0.0115 (14)
C48A	0.0213 (14)	0.0253 (15)	0.0271 (15)	0.0016 (12)	−0.0102 (12)	−0.0069 (12)
C49	0.0402 (18)	0.0253 (16)	0.0243 (15)	0.0042 (13)	−0.0101 (13)	−0.0101 (12)
C50	0.0445 (19)	0.0248 (16)	0.0234 (15)	0.0066 (13)	−0.0119 (14)	−0.0078 (12)
C50A	0.0303 (16)	0.0240 (15)	0.0307 (16)	0.0082 (12)	−0.0114 (13)	−0.0140 (13)
C411	0.0422 (19)	0.0222 (16)	0.0370 (18)	0.0044 (13)	−0.0106 (15)	−0.0121 (13)
C412	0.0348 (19)	0.044 (2)	0.068 (2)	0.0086 (15)	−0.0116 (17)	−0.0356 (18)
C413	0.056 (2)	0.047 (2)	0.076 (3)	0.0144 (18)	−0.037 (2)	−0.033 (2)
C414	0.0292 (17)	0.0363 (17)	0.0450 (19)	0.0095 (13)	−0.0147 (14)	−0.0169 (15)
C415	0.068 (2)	0.0279 (18)	0.043 (2)	0.0154 (16)	−0.0241 (18)	−0.0126 (15)
C416	0.053 (2)	0.0322 (19)	0.064 (3)	−0.0053 (16)	0.0056 (19)	−0.0148 (18)

### Geometric parameters (Å, °)

**Table:**

O1—C7	1.384 (3)	O3—C37	1.378 (4)
O1—H1	0.8400	O3—H3	0.8400
C1—C2	1.524 (4)	C31—C32	1.528 (4)
C1—C15	1.532 (4)	C31—C316	1.530 (4)
C1—C16	1.543 (4)	C31—C315	1.544 (4)
C1—C10A	1.559 (4)	C31—C40A	1.553 (4)
C2—C3	1.512 (4)	C32—C33	1.501 (4)
C2—H2A	0.9900	C32—H32A	0.9900
C2—H2B	0.9900	C32—H32B	0.9900
C3—C4	1.526 (4)	C33—C34	1.527 (4)
C3—H3A	0.9900	C33—H33A	0.9900
C3—H3B	0.9900	C33—H33B	0.9900
C4—C4A	1.538 (4)	C34—C34A	1.537 (4)
C4—H4A	0.9900	C34—H34A	0.9900
C4—H4B	0.9900	C34—H34B	0.9900
C4A—C14	1.533 (4)	C34A—C34B	1.537 (4)
C4A—C4B	1.537 (4)	C34A—C314	1.541 (4)
C4A—C10A	1.547 (4)	C34A—C40A	1.545 (4)
C4B—C5	1.385 (4)	C34B—C35	1.387 (4)
C4B—C8A	1.395 (4)	C34B—C38A	1.398 (4)
C5—C6	1.363 (4)	C35—C36	1.369 (4)
C5—H5	0.9500	C35—H35	0.9500
C6—C7	1.369 (4)	C36—C37	1.367 (5)
C6—H6	0.9500	C36—H36	0.9500
C7—C8	1.394 (4)	C37—C38	1.382 (4)
C8—C8A	1.406 (4)	C38—C38A	1.409 (4)
C8—C11	1.521 (4)	C38—C311	1.523 (4)
C8A—C9	1.511 (4)	C38A—C39	1.517 (4)
C9—C10	1.526 (4)	C39—C40	1.528 (4)
C9—H9A	0.9900	C39—H39A	0.9900
C9—H9B	0.9900	C39—H39B	0.9900
C10—C10A	1.517 (4)	C40A—C40	1.524 (4)
C10—H10B	0.9900	C40A—H40A	1.0000
C10—H10C	0.9900	C40—H40B	0.9900
C10A—H10A	1.0000	C40—H40C	0.9900
C11—C12	1.518 (4)	C311—C312	1.499 (5)
C11—C13	1.522 (4)	C311—C313	1.522 (4)
C11—H11	1.0000	C311—H311	1.0000
C12—H12A	0.9800	C312—H12G	0.9800
C12—H12B	0.9800	C312—H12H	0.9800
C12—H12C	0.9800	C312—H12I	0.9800
C13—H13A	0.9800	C313—H13G	0.9800
C13—H13B	0.9800	C313—H13H	0.9800
C13—H13C	0.9800	C313—H13I	0.9800
C14—H14A	0.9800	C314—H14G	0.9800
C14—H14B	0.9800	C314—H14H	0.9800
C14—H14C	0.9800	C314—H14I	0.9800
C15—H15A	0.9800	C315—H15G	0.9800
C15—H15B	0.9800	C315—H15H	0.9800
C15—H15C	0.9800	C315—H15I	0.9800
C16—H16A	0.9800	C316—H16G	0.9800
C16—H16B	0.9800	C316—H16H	0.9800
C16—H16C	0.9800	C316—H16I	0.9800
O2—C27	1.384 (3)	O4—C47	1.394 (3)
O2—H2	0.8400	O4—H4	0.8400
C21—C22	1.532 (4)	C41—C415	1.526 (4)
C21—C216	1.534 (4)	C41—C42	1.531 (4)
C21—C215	1.550 (4)	C41—C416	1.535 (4)
C21—C30A	1.557 (4)	C41—C50A	1.557 (4)
C22—C23	1.507 (4)	C42—C43	1.518 (5)
C22—H22A	0.9900	C42—H42A	0.9900
C22—H22B	0.9900	C42—H42B	0.9900
C23—C24	1.527 (4)	C43—C44	1.523 (4)
C23—H23A	0.9900	C43—H43A	0.9900
C23—H23B	0.9900	C43—H43B	0.9900
C24—C24A	1.544 (4)	C44—C44A	1.532 (4)
C24—H24A	0.9900	C44—H44A	0.9900
C24—H24B	0.9900	C44—H44B	0.9900
C24A—C24B	1.535 (4)	C44A—C44B	1.535 (4)
C24A—C214	1.541 (4)	C44A—C414	1.539 (4)
C24A—C30A	1.549 (4)	C44A—C50A	1.543 (4)
C24B—C25	1.393 (4)	C44B—C45	1.387 (4)
C24B—C28A	1.403 (4)	C44B—C48A	1.400 (4)
C25—C26	1.364 (4)	C45—C46	1.381 (4)
C25—H25	0.9500	C45—H45	0.9500
C26—C27	1.370 (4)	C46—C47	1.362 (4)
C26—H26	0.9500	C46—H46	0.9500
C27—C28	1.378 (4)	C47—C48	1.382 (4)
C28—C28A	1.414 (4)	C48—C48A	1.412 (4)
C28—C211	1.519 (4)	C48—C411	1.530 (4)
C28A—C29	1.506 (4)	C48A—C49	1.509 (4)
C29—C30	1.525 (4)	C49—C50	1.517 (4)
C29—H29A	0.9900	C49—H49A	0.9900
C29—H29B	0.9900	C49—H49B	0.9900
C30—C30A	1.527 (4)	C50—C50A	1.515 (4)
C30—H30B	0.9900	C50—H50B	0.9900
C30—H30C	0.9900	C50—H50C	0.9900
C30A—H30A	1.0000	C50A—H50A	1.0000
C211—C213	1.514 (4)	C411—C412	1.517 (4)
C211—C212	1.517 (4)	C411—C413	1.530 (4)
C211—H211	1.0000	C411—H411	1.0000
C212—H12D	0.9800	C412—H12J	0.9800
C212—H12E	0.9800	C412—H12K	0.9800
C212—H12F	0.9800	C412—H12L	0.9800
C213—H13D	0.9800	C413—H13J	0.9800
C213—H13E	0.9800	C413—H13K	0.9800
C213—H13F	0.9800	C413—H13L	0.9800
C214—H14D	0.9800	C414—H14J	0.9800
C214—H14E	0.9800	C414—H14K	0.9800
C214—H14F	0.9800	C414—H14L	0.9800
C215—H15D	0.9800	C415—H15J	0.9800
C215—H15E	0.9800	C415—H15K	0.9800
C215—H15F	0.9800	C415—H15L	0.9800
C216—H16D	0.9800	C416—H16J	0.9800
C216—H16E	0.9800	C416—H16K	0.9800
C216—H16F	0.9800	C416—H16L	0.9800
			
C7—O1—H1	109.5	C37—O3—H3	109.5
C2—C1—C15	110.6 (2)	C32—C31—C316	107.3 (3)
C2—C1—C16	107.2 (3)	C32—C31—C315	109.8 (3)
C15—C1—C16	107.0 (3)	C316—C31—C315	107.6 (3)
C2—C1—C10A	108.4 (2)	C32—C31—C40A	108.6 (2)
C15—C1—C10A	114.7 (2)	C316—C31—C40A	108.5 (2)
C16—C1—C10A	108.8 (2)	C315—C31—C40A	114.8 (2)
C3—C2—C1	113.4 (2)	C33—C32—C31	113.9 (3)
C3—C2—H2A	108.9	C33—C32—H32A	108.8
C1—C2—H2A	108.9	C31—C32—H32A	108.8
C3—C2—H2B	108.9	C33—C32—H32B	108.8
C1—C2—H2B	108.9	C31—C32—H32B	108.8
H2A—C2—H2B	107.7	H32A—C32—H32B	107.7
C2—C3—C4	111.3 (2)	C32—C33—C34	110.5 (3)
C2—C3—H3A	109.4	C32—C33—H33A	109.6
C4—C3—H3A	109.4	C34—C33—H33A	109.6
C2—C3—H3B	109.4	C32—C33—H33B	109.6
C4—C3—H3B	109.4	C34—C33—H33B	109.6
H3A—C3—H3B	108.0	H33A—C33—H33B	108.1
C3—C4—C4A	112.6 (2)	C33—C34—C34A	112.1 (2)
C3—C4—H4A	109.1	C33—C34—H34A	109.2
C4A—C4—H4A	109.1	C34A—C34—H34A	109.2
C3—C4—H4B	109.1	C33—C34—H34B	109.2
C4A—C4—H4B	109.1	C34A—C34—H34B	109.2
H4A—C4—H4B	107.8	H34A—C34—H34B	107.9
C14—C4A—C4B	107.0 (2)	C34B—C34A—C34	110.9 (2)
C14—C4A—C4	109.1 (2)	C34B—C34A—C314	106.3 (2)
C4B—C4A—C4	110.2 (2)	C34—C34A—C314	108.9 (2)
C14—C4A—C10A	114.9 (2)	C34B—C34A—C40A	107.8 (2)
C4B—C4A—C10A	107.8 (2)	C34—C34A—C40A	107.9 (2)
C4—C4A—C10A	107.8 (2)	C314—C34A—C40A	115.0 (2)
C5—C4B—C8A	117.9 (3)	C35—C34B—C38A	118.2 (3)
C5—C4B—C4A	118.7 (2)	C35—C34B—C34A	119.6 (3)
C8A—C4B—C4A	123.4 (2)	C38A—C34B—C34A	122.2 (2)
C6—C5—C4B	121.9 (3)	C36—C35—C34B	121.1 (3)
C6—C5—H5	119.0	C36—C35—H35	119.5
C4B—C5—H5	119.0	C34B—C35—H35	119.5
C5—C6—C7	119.9 (3)	C37—C36—C35	120.5 (3)
C5—C6—H6	120.0	C37—C36—H36	119.7
C7—C6—H6	120.0	C35—C36—H36	119.7
C6—C7—O1	120.7 (3)	C36—C37—O3	121.1 (3)
C6—C7—C8	121.1 (3)	C36—C37—C38	121.1 (3)
O1—C7—C8	118.1 (3)	O3—C37—C38	117.8 (3)
C7—C8—C8A	117.9 (3)	C37—C38—C38A	118.2 (3)
C7—C8—C11	120.3 (3)	C37—C38—C311	120.6 (3)
C8A—C8—C11	121.9 (3)	C38A—C38—C311	121.2 (3)
C4B—C8A—C8	121.1 (2)	C34B—C38A—C38	120.9 (3)
C4B—C8A—C9	120.6 (2)	C34B—C38A—C39	120.6 (2)
C8—C8A—C9	118.3 (2)	C38—C38A—C39	118.5 (3)
C8A—C9—C10	114.2 (2)	C38A—C39—C40	115.6 (2)
C8A—C9—H9A	108.7	C38A—C39—H39A	108.4
C10—C9—H9A	108.7	C40—C39—H39A	108.4
C8A—C9—H9B	108.7	C38A—C39—H39B	108.4
C10—C9—H9B	108.7	C40—C39—H39B	108.4
H9A—C9—H9B	107.6	H39A—C39—H39B	107.4
C10A—C10—C9	109.5 (2)	C40—C40A—C34A	108.8 (2)
C10A—C10—H10B	109.8	C40—C40A—C31	113.1 (2)
C9—C10—H10B	109.8	C34A—C40A—C31	118.1 (2)
C10A—C10—H10C	109.8	C40—C40A—H40A	105.2
C9—C10—H10C	109.8	C34A—C40A—H40A	105.2
H10B—C10—H10C	108.2	C31—C40A—H40A	105.2
C10—C10A—C4A	109.0 (2)	C40A—C40—C39	110.7 (2)
C10—C10A—C1	115.4 (2)	C40A—C40—H40B	109.5
C4A—C10A—C1	116.9 (2)	C39—C40—H40B	109.5
C10—C10A—H10A	104.7	C40A—C40—H40C	109.5
C4A—C10A—H10A	104.7	C39—C40—H40C	109.5
C1—C10A—H10A	104.7	H40B—C40—H40C	108.1
C12—C11—C8	113.4 (3)	C312—C311—C313	110.1 (3)
C12—C11—C13	109.8 (3)	C312—C311—C38	112.7 (3)
C8—C11—C13	112.9 (3)	C313—C311—C38	114.3 (3)
C12—C11—H11	106.8	C312—C311—H311	106.4
C8—C11—H11	106.8	C313—C311—H311	106.4
C13—C11—H11	106.8	C38—C311—H311	106.4
C11—C12—H12A	109.5	C311—C312—H12G	109.5
C11—C12—H12B	109.5	C311—C312—H12H	109.5
H12A—C12—H12B	109.5	H12G—C312—H12H	109.5
C11—C12—H12C	109.5	C311—C312—H12I	109.5
H12A—C12—H12C	109.5	H12G—C312—H12I	109.5
H12B—C12—H12C	109.5	H12H—C312—H12I	109.5
C11—C13—H13A	109.5	C311—C313—H13G	109.5
C11—C13—H13B	109.5	C311—C313—H13H	109.5
H13A—C13—H13B	109.5	H13G—C313—H13H	109.5
C11—C13—H13C	109.5	C311—C313—H13I	109.5
H13A—C13—H13C	109.5	H13G—C313—H13I	109.5
H13B—C13—H13C	109.5	H13H—C313—H13I	109.5
C4A—C14—H14A	109.5	C34A—C314—H14G	109.5
C4A—C14—H14B	109.5	C34A—C314—H14H	109.5
H14A—C14—H14B	109.5	H14G—C314—H14H	109.5
C4A—C14—H14C	109.5	C34A—C314—H14I	109.5
H14A—C14—H14C	109.5	H14G—C314—H14I	109.5
H14B—C14—H14C	109.5	H14H—C314—H14I	109.5
C1—C15—H15A	109.5	C31—C315—H15G	109.5
C1—C15—H15B	109.5	C31—C315—H15H	109.5
H15A—C15—H15B	109.5	H15G—C315—H15H	109.5
C1—C15—H15C	109.5	C31—C315—H15I	109.5
H15A—C15—H15C	109.5	H15G—C315—H15I	109.5
H15B—C15—H15C	109.5	H15H—C315—H15I	109.5
C1—C16—H16A	109.5	C31—C316—H16G	109.5
C1—C16—H16B	109.5	C31—C316—H16H	109.5
H16A—C16—H16B	109.5	H16G—C316—H16H	109.5
C1—C16—H16C	109.5	C31—C316—H16I	109.5
H16A—C16—H16C	109.5	H16G—C316—H16I	109.5
H16B—C16—H16C	109.5	H16H—C316—H16I	109.5
C27—O2—H2	109.5	C47—O4—H4	109.5
C22—C21—C216	107.1 (2)	C415—C41—C42	110.9 (2)
C22—C21—C215	111.1 (3)	C415—C41—C416	107.8 (3)
C216—C21—C215	106.9 (3)	C42—C41—C416	106.7 (3)
C22—C21—C30A	108.3 (2)	C415—C41—C50A	114.3 (3)
C216—C21—C30A	108.9 (2)	C42—C41—C50A	108.3 (2)
C215—C21—C30A	114.3 (2)	C416—C41—C50A	108.6 (2)
C23—C22—C21	113.6 (2)	C43—C42—C41	113.8 (3)
C23—C22—H22A	108.9	C43—C42—H42A	108.8
C21—C22—H22A	108.9	C41—C42—H42A	108.8
C23—C22—H22B	108.9	C43—C42—H42B	108.8
C21—C22—H22B	108.9	C41—C42—H42B	108.8
H22A—C22—H22B	107.7	H42A—C42—H42B	107.7
C22—C23—C24	111.2 (3)	C42—C43—C44	110.8 (3)
C22—C23—H23A	109.4	C42—C43—H43A	109.5
C24—C23—H23A	109.4	C44—C43—H43A	109.5
C22—C23—H23B	109.4	C42—C43—H43B	109.5
C24—C23—H23B	109.4	C44—C43—H43B	109.5
H23A—C23—H23B	108.0	H43A—C43—H43B	108.1
C23—C24—C24A	111.8 (2)	C43—C44—C44A	112.0 (2)
C23—C24—H24A	109.3	C43—C44—H44A	109.2
C24A—C24—H24A	109.3	C44A—C44—H44A	109.2
C23—C24—H24B	109.3	C43—C44—H44B	109.2
C24A—C24—H24B	109.3	C44A—C44—H44B	109.2
H24A—C24—H24B	107.9	H44A—C44—H44B	107.9
C24B—C24A—C214	107.2 (2)	C44—C44A—C44B	110.5 (2)
C24B—C24A—C24	110.5 (2)	C44—C44A—C414	108.1 (2)
C214—C24A—C24	108.5 (2)	C44B—C44A—C414	106.1 (2)
C24B—C24A—C30A	107.4 (2)	C44—C44A—C50A	108.9 (2)
C214—C24A—C30A	115.1 (2)	C44B—C44A—C50A	107.9 (2)
C24—C24A—C30A	108.1 (2)	C414—C44A—C50A	115.3 (2)
C25—C24B—C28A	117.9 (3)	C45—C44B—C48A	117.9 (3)
C25—C24B—C24A	119.4 (3)	C45—C44B—C44A	119.5 (2)
C28A—C24B—C24A	122.7 (2)	C48A—C44B—C44A	122.5 (2)
C26—C25—C24B	121.6 (3)	C46—C45—C44B	121.9 (3)
C26—C25—H25	119.2	C46—C45—H45	119.0
C24B—C25—H25	119.2	C44B—C45—H45	119.0
C25—C26—C27	120.0 (3)	C47—C46—C45	119.2 (3)
C25—C26—H26	120.0	C47—C46—H46	120.4
C27—C26—H26	120.0	C45—C46—H46	120.4
C26—C27—C28	121.9 (3)	C46—C47—C48	122.1 (3)
C26—C27—O2	120.1 (3)	C46—C47—O4	119.5 (3)
C28—C27—O2	118.0 (3)	C48—C47—O4	118.4 (3)
C27—C28—C28A	117.9 (3)	C47—C48—C48A	118.1 (3)
C27—C28—C211	121.0 (3)	C47—C48—C411	120.2 (3)
C28A—C28—C211	121.1 (2)	C48A—C48—C411	121.6 (2)
C24B—C28A—C28	120.8 (2)	C44B—C48A—C48	120.6 (2)
C24B—C28A—C29	120.7 (2)	C44B—C48A—C49	120.8 (2)
C28—C28A—C29	118.5 (2)	C48—C48A—C49	118.5 (2)
C28A—C29—C30	115.3 (2)	C48A—C49—C50	114.7 (2)
C28A—C29—H29A	108.5	C48A—C49—H49A	108.6
C30—C29—H29A	108.5	C50—C49—H49A	108.6
C28A—C29—H29B	108.5	C48A—C49—H49B	108.6
C30—C29—H29B	108.5	C50—C49—H49B	108.6
H29A—C29—H29B	107.5	H49A—C49—H49B	107.6
C29—C30—C30A	109.3 (2)	C50A—C50—C49	110.4 (2)
C29—C30—H30B	109.8	C50A—C50—H50B	109.6
C30A—C30—H30B	109.8	C49—C50—H50B	109.6
C29—C30—H30C	109.8	C50A—C50—H50C	109.6
C30A—C30—H30C	109.8	C49—C50—H50C	109.6
H30B—C30—H30C	108.3	H50B—C50—H50C	108.1
C30—C30A—C24A	109.0 (2)	C50—C50A—C44A	108.9 (2)
C30—C30A—C21	113.8 (2)	C50—C50A—C41	114.3 (2)
C24A—C30A—C21	117.8 (2)	C44A—C50A—C41	117.4 (2)
C30—C30A—H30A	105.0	C50—C50A—H50A	105.0
C24A—C30A—H30A	105.0	C44A—C50A—H50A	105.0
C21—C30A—H30A	105.0	C41—C50A—H50A	105.0
C213—C211—C212	110.1 (2)	C412—C411—C48	112.6 (3)
C213—C211—C28	113.4 (2)	C412—C411—C413	110.3 (3)
C212—C211—C28	112.7 (2)	C48—C411—C413	112.6 (3)
C213—C211—H211	106.7	C412—C411—H411	107.0
C212—C211—H211	106.7	C48—C411—H411	107.0
C28—C211—H211	106.7	C413—C411—H411	107.0
C211—C212—H12D	109.5	C411—C412—H12J	109.5
C211—C212—H12E	109.5	C411—C412—H12K	109.5
H12D—C212—H12E	109.5	H12J—C412—H12K	109.5
C211—C212—H12F	109.5	C411—C412—H12L	109.5
H12D—C212—H12F	109.5	H12J—C412—H12L	109.5
H12E—C212—H12F	109.5	H12K—C412—H12L	109.5
C211—C213—H13D	109.5	C411—C413—H13J	109.5
C211—C213—H13E	109.5	C411—C413—H13K	109.5
H13D—C213—H13E	109.5	H13J—C413—H13K	109.5
C211—C213—H13F	109.5	C411—C413—H13L	109.5
H13D—C213—H13F	109.5	H13J—C413—H13L	109.5
H13E—C213—H13F	109.5	H13K—C413—H13L	109.5
C24A—C214—H14D	109.5	C44A—C414—H14J	109.5
C24A—C214—H14E	109.5	C44A—C414—H14K	109.5
H14D—C214—H14E	109.5	H14J—C414—H14K	109.5
C24A—C214—H14F	109.5	C44A—C414—H14L	109.5
H14D—C214—H14F	109.5	H14J—C414—H14L	109.5
H14E—C214—H14F	109.5	H14K—C414—H14L	109.5
C21—C215—H15D	109.5	C41—C415—H15J	109.5
C21—C215—H15E	109.5	C41—C415—H15K	109.5
H15D—C215—H15E	109.5	H15J—C415—H15K	109.5
C21—C215—H15F	109.5	C41—C415—H15L	109.5
H15D—C215—H15F	109.5	H15J—C415—H15L	109.5
H15E—C215—H15F	109.5	H15K—C415—H15L	109.5
C21—C216—H16D	109.5	C41—C416—H16J	109.5
C21—C216—H16E	109.5	C41—C416—H16K	109.5
H16D—C216—H16E	109.5	H16J—C416—H16K	109.5
C21—C216—H16F	109.5	C41—C416—H16L	109.5
H16D—C216—H16F	109.5	H16J—C416—H16L	109.5
H16E—C216—H16F	109.5	H16K—C416—H16L	109.5

### Table 1 Puckering amplitudes (Cremer &amp; Pople, 1975) for the unsaturated six-membered rings within the four independent molecules

**Table:**

	C1/C2/C3/C4/C4a/C10a			C4a/C4b/C8a/C9/C10/C10a		
Molecule	Q (Å)	θ (°)	φ (°)	Q (Å)	θ (°)	φ (°)
1	0.553 (3)	4.2 (3)	140 (5)	0.553 (3)	52.3 (3)	284.7 (4)
2	0.550 (3)	6.8 (3)	141 (3)	0.555 (3)	51.9 (3)	289.8 (4)
3	0.548 (4)	7.9 (4)	142 (3)	0.543 (3)	51.0 (3)	296.2 (4)
4	0.547 (4)	6.6 (4)	139 (3)	0.543 (3)	51.0 (3)	289.2 (4)

### Table 2 Structure matching between the four independent molecules

A is the structure match between molecules 1 and 2, B is that between molecules 1 and 3, C is that between molecules 1 and 4, D is that between molecules 2 and 3, E is that between molecules 2 and 4 and F is that between molecules 3 and 4.

**Table:**

Overlay	r.m.s. position (Å)	r.m.s. bond (Å)	r.m.s. torsion (°)
A	0.0707	0.0074	2.1002
B	0.1754	0.0074	5.0807
C	0.0917	0.0073	2.7283
D	0.1174	0.0063	3.479
E	0.0505	0.0093	1.5649
F	0.0896	0.0091	2.6273
